# How do preschoolers and adults ascribe authority?

**DOI:** 10.1016/j.isci.2025.113279

**Published:** 2025-08-07

**Authors:** Sarah Pieper, Sara Weber, Anna Neuwerk, Sarah Tune, Sarah Jessen

**Affiliations:** 1 Center of Brain, Behavior, and Metabolism, University of Lübeck, 23562 Lübeck, Germany; 2 Institute of Psychology, University of Lübeck, 23562 Lübeck, Germany; 3 Institute of Medical Psychology, University of Lübeck, 23562 Lübeck, Germany; 4 Department of Psychiatry and Psychotherapy, University of Lübeck, 23562 Lübeck, Germany

**Keywords:** Social sciences, Research methodology social sciences

## Abstract

Determining social hierarchies is an essential part of successful social behavior and already children are aware of hierarchical relationships. However, which cues humans use to determine hierarchies is highly variable; it includes behavioral as well as perceptual cues and changes throughout development. To investigate the interplay between different cues, preschoolers and adults participated in a behavioral paradigm comparing the impact of helping behavior (behavioral cue) and body height (perceptual cue) on the attribution of authority. Results revealed a double dissociation: Children did not use helping behavior as an indicator but attributed more authority to taller individuals. In contrast, adults ascribed more authority to a person who refused to help, but did not consider body height. A helping person was generally judged as nicer, suggesting that both, children and adults, interpreted the depicted situations correctly. Hence, our results suggest that children and adults use different information to attribute authority.

## Highlights

• Preschoolers and adults successfully navigate social hierarchies
• Preschoolers used body height as a cue for authority
• Adults used refusal to help as a cue for authority
• Both, preschoolers and adults, judge a helping actress as nicer

## Graphical abstract

**Figure:**
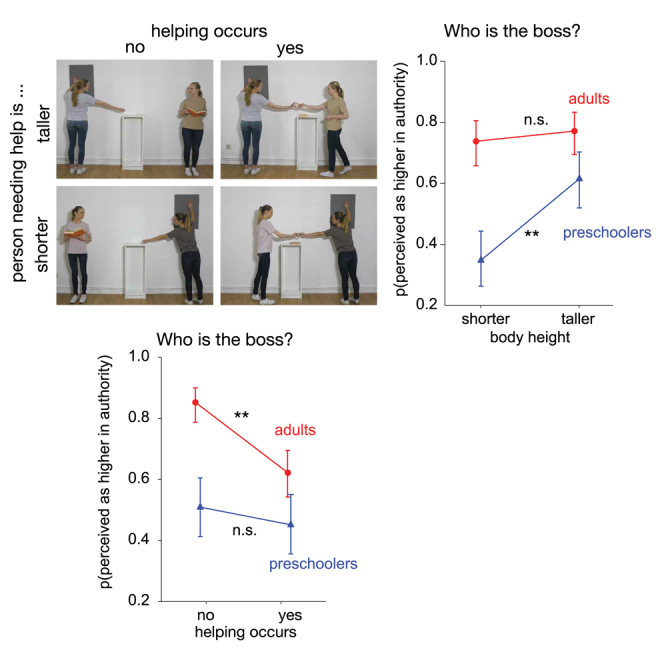

## Introduction

From birth, humans live in social structures. These social structures influence almost every aspect of our lives and are often hierarchical in nature; individuals differ in how much authority they carry over others. To successfully navigate social structures, humans need an accurate understanding of these hierarchies and the concomitant differences in authority, which defines the relationships between individuals and their hierarchical position to each other.^1^ Sometimes, these positions are labeled explicitly (“She is the boss.”), but most often social hierarchies are more implicit and need to be inferred.

Human adults use a number of different cues to attribute authority to a person. Some of these cues are rooted in overt behavior. Adults, for instance, attribute more authority to individuals who give orders or grant (or deny) permission.^2^ In addition, adults use less specific behavioral cues such as body posture and general demeanor; a person speaking loudly with an erect posture and broad gestures is typically perceived as higher in authority compared to a person with a slouched position and a soft voice; for recent review, see Carney (2020).^3^ Besides these behavioral cues, several studies suggest that attribution of hierarchy is also influenced by outer appearance. Such cues include facial features (e.g., Oosterhof & Todorov, 2008^4^) and physical strength (e.g., Lukaszewski et al., 2016^5^).

In sum, adults show a sophisticated system of determining authority via different cues, often used in combination. However, social hierarchies not only impact the lives of adults but are relevant from early on in ontogeny. Hence, it is not surprising that a sensitivity to social hierarchies can already be observed in early development.

Already at one year of age, infants expect smaller individuals to be submissive to larger ones.^6^ Furthermore, infants at 17 months of age expect individuals who are higher in power to command more resources compared to individuals who are lower in power.^7^ As children grow older, their understanding of cues signaling authority becomes more sophisticated. For instance, between 3 and 5 years of age, children start to attribute more authority to people exhibiting more expansive body postures.^8^^,^^9^

Starting in the second year of life, children become not only sensitive to power relations in a given situation but expect difference in power to be stable across situations,^1^^,^^10^ a fundamental prerequisite for determining social hierarchies. By the age of five, a temporally stable preference for more socially powerful individuals is evident: children are significantly more likely to imitate the behavior of adults with high social status than those with low social status.^11^^,^^12^ By the age of six, children can make temporally stable predictions about individuals’ behavior depending on their social power.^13^ An adult-like concept of social power can be expected between the ages of seven and nine years.^2^^,^^8^

While we know that numerous hierarchy cues are effectively used in development, we know little about the interplay between different factors when more than one cue is available. The goal of the present study is therefore to investigate the contribution of two cues, namely body height (a perceptual cue) and refusal of prosocial behavior (a behavioral cue), to the perception of social hierarchy in children and adults.

### Prosocial behavior and body size as a hierarchy cue

The term “prosocial behavior” can be defined as interpersonal behavior that follows the purpose of serving the well-being of another person^14^ and covers a wide range of behaviors. The current study focuses on one facet of prosocial behavior, namely helping behavior, as an example of prosocial behavior that is already shown by young children^15^ and has been used successfully in comparable studies investigating the impact of prosocial cues on perception of hierarchy in children.^13^

A number of previous studies have investigated the interplay between social power and prosocial behavior, yielding somewhat contradicting findings. On the one hand, children and adults with low social status show more prosocial behavior than individuals with high social status.^16^^,^^17^ These results are consistent with the findings of Galinsky et al., 2006,^18^ who suggest that socially powerful individuals are less able to empathize with the thoughts, perceptions, and feelings of others. On the other hand, several studies report the opposite pattern. In these studies, individuals with higher social status show more prosocial behavior, which could be seen as a strategy to maintain their high social status.^19^ This is also supported by the noblesse oblige concept, which states that the social norm encourages individuals with high social status to behave particularly generously toward less privileged individuals.^20^ One explanation for these discrepant findings might arise from the different research approaches used; while many studies reporting less prosocial behavior in high status individuals report results from lab-based experiments, studies reporting more prosocial behavior in high status individuals often use large-scale observational studies.^21^

A similar ambiguous pattern has been observed in children, where socially powerful children show both more prosocial behavior but also exertion of coercion toward their playmates.^22^ In experimental settings, Terrizzi et al., 2020^13^ systematically investigated the influence of helping behavior on ascription of authority in children and adults. In their study, 4–7-year-old children as well as adults watched videos in which a person unsuccessfully tried to perform an action (an actress tried to mount a poster on a wall but could not reach the tape). A second person observed the action and either helped the other person or ignored their need. While participants made no consistent judgment of authority in the helping scenario, participants of all age groups judged a person who refused help as higher in authority compared to a person in need of help.^13^

In contrast to helping behavior, body size is a physical cue relying purely on perceptual features. Relative body size influences the assessment of social power in infants,^6^ children,^23^^,^^24^ and adults.^23^^,^^25^ Similarly, relative body size also influences the selection of helpers. Children as young as three-and-a-half years old are more likely to select stronger individuals as helpers, especially in tasks that require physical strength.^26^ Interestingly, the relationship between perceived height and authority also works the other way around; individuals are perceived as taller if they exhibit a demeanor typically associated with authority.^25^ While at least in adults, physical prowess is rarely needed to exert authority, some studies also suggest a link between height and position in social hierarchy (e.g., Judge & Cable, 2004^27^ and Stulp et al., 2012^28^).

In sum, both prosocial behavior and body size appear to influence ascription of authority. While the direction of influence is clear for body size —larger individuals tend to be perceived as higher in authority— the link with prosocial behavior is more complex. Importantly, in real life, cues to social hierarchy rarely occur in isolation, but typically multiple, sometimes conflicting, cues are present at the same time. The question therefore arises, which of those cues —body height or prosocial behavior— drives the ascription of authority if both are present simultaneously and provide conflicting information.

### Prosocial behavior as a cue for niceness

Besides serving as a cue for hierarchical positions, prosocial behavior impacts our impression of another person in several other ways. One prominent effect of prosocial behavior is that a person behaving prosocial is perceived as nicer by both children and adults (e.g., Terrizzi et al., 2020^13^). We therefore decided to assess perceived niceness as a control variable, ensuring children (and adults) understood the content of the stimulus material and judged a person who provided help as nicer compared to a person who refused to help. Such an attribution has been repeatedly observed in the past in infants^29^ as well as children and adults.^13^

### The current study

**Figure 1:**
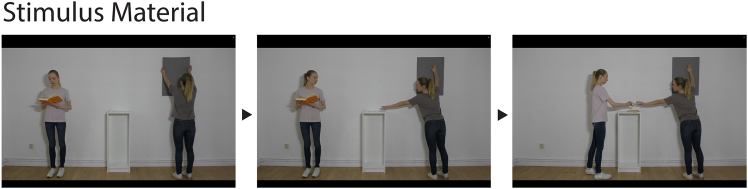
Example of stimulus material used Shown are three frames from one of the four stimulus videos. The index person (wearing the white t-shirt) is taller than the other actress (wearing the gray shirt) and provides help by passing the tape. In the condition in which help is refused, the index person also looks at the other actress (as shown in the middle frame) but then returns to reading her book.

**Figure 2:**
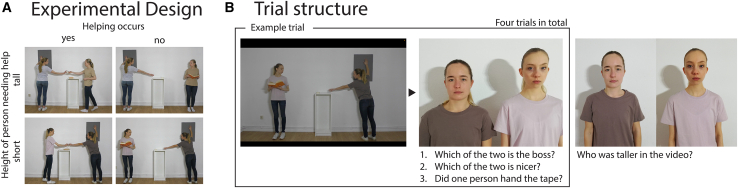
Overview of the experiment (A) Experimental Design. In a 2 × 2 within-subject design, we manipulated the factors Helping and Body Height. For each condition, one video was created. (B) Trial structure. All four videos (one per condition) were shown to the participants and after each video, the participant was asked the same three questions while both actresses were shown on the screen. After the four videos, the actresses were shown again, pairwise aligned so that their shoulders did not differ in height and the participant was asked who had been taller in the previous videos.

In sum, the current study examines the interplay of prosocial behavior and relative body height in the perception of social power and niceness in one study design. To do so, adults and preschoolers watched short video clips in which an actress either helped another one or refused to do so (Figure 1), and participants were asked to judge who is the boss and who is nicer (Figure 2). In addition, one of the actresses was always taller than the other. Based on previous findings by Terrizzi et al., 2020,^13^ we expected individuals who refused to help to be judged as higher in social hierarchy by both children and adults (Hypothesis 1). Furthermore, we expected both children and adults to perceive taller individuals as higher in authority compared to shorter individuals (see e.g., Lourenco et al., 2015^23^; Hypothesis 2). Finally, we expected both children and adults to perceive helping individuals as nicer compared to non-helping individuals (e.g., Terrizzi et al., 2020^13^; Hypothesis 3).

## Results

### Adults, but not children, associate prosocial behavior with lower authority

**Figure 3:**
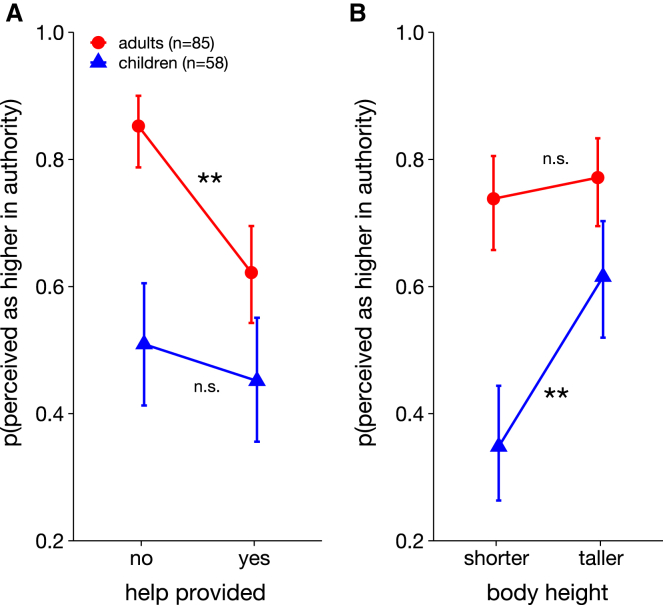
Perceived authority as a function of social behavior and body height (A-B) Model-predicted marginal means of perceived authority as a function of observed social behavior (A) and body height (B) of the index person. As revealed by significant two-way interactions of helping and body height with age group, we observed a double dissociation of how children (blue triangle and line) and adults (red circle and line) weighted the behavioral and the perceptual cue in their perception of authority. Error bars show the 95% confidence interval. n.s. = not significant; ∗∗ = *p* <0 .001.

The statistical analysis of perceived authority revealed that individuals who provide help were generally judged to be lower in social hierarchy, that is, they were less likely to be perceived as the boss (odds ratio (OR) = 0.48, 95% confidence interval (CI) [0.33, 0.70], *p* < 0.001, see Table S1 for full model details and Table 1 for model-derived results, respectively). However, as the significant interaction with Age group showed (OR = 2.8, CI [1.3,5.9], *p* = 0.008), this effect was driven by the adult participants only: In the adult sample, we observed a significant main effect of Help (OR = 0.27, CI[0.15,0.47], *p* < 0.001, see Figure 3A; Table S7), whereas this effect was absent in the children sample (OR = 0.80, CI[0.46,1.4], *p* = 0.41, Bayes Factor (BF_01_) = 11.2). In other words, under statistical control of all other modeled influences, the probability of adult participants perceiving the index person as higher in authority increased from 62% when providing help to 85% when she refused to help (see Figure 3A). In contrast, the Bayes factor observed in the children sample suggests that our findings provide positive evidence in favor of the null hypothesis (i.e., no influence of prosocial behavior on ascription of authority) as the data are 11.2 times more likely under the null hypothesis than under the alternative hypothesis. Finally, adults (compared to children) perceived the index person as higher in authority, irrespective of her behavior (OR = 0.3 CI [0.2–0.45], *p* < 0.001).

**Table 1:** Condition-specific model-predicted probabilities and relative risks for helping behavior and body height and ascription of authority

Predicted probabilities and relative risks				
*Help*	*Height*	*Adults p (95% CI)*	*Children p (95% CI)*	*RR (Children/Adults; 95% CI)*
No	shorter	0.853 (0.758, 0.914)	0.394 (0.274, 0.527)	0.462 (0.328, 0.650)
Yes	shorter	0.579 (0.469, 0.682)	0.305 (0.198, 0.438)	0.528 (0.339, 0.821)
No	taller	0.853 (0.758, 0.914)	0.624 (0.490, 0.742)	0.732 (0.586, 0.915)
Yes	taller	0.663 (0.553, 0.758)	0.606 (0.473, 0.726)	0.914 (0.703, 1.190)

The model for the full sample was able to explain 17.5% of the variance using fixed effects (R^2^_marginal_ = 0.175) and 20.5% using fixed and random effects (R^2^_conditional_ = 0.205). Note that while these values might appear rather small, comparable effect sizes are common and meaningful in social sciences.^30^ The intraclass correlation (ICC) shows that 3.6% of the total variance can be attributed to the variance between subjects (ICC = 0.0360).

In an additional control analysis, we tested whether behavioral cue usage changed with age in the sample of preschool children. However, the inclusion of continuously measured age as a main effect and in interaction with our cue manipulation did not yield any additional significant effects (all *p*-values >0.43). Importantly, when excluding all participants who had failed to correctly answer at least five of the method check questions (*N* = 9), our main finding of divergent cue-usage for adults vs. children remained qualitatively unchanged (see Tables S2 and S3).

Thus, while adults associated the refusal to help with authority, this effect was not found in the preschoolers. The results of^13^ regarding the first hypothesis could therefore only be replicated in the sample of adult participants but not in the preschooler sample.

### Children, but not adults, base authority judgements on body height

Next, we tested our second hypothesis that taller individuals should be judged as higher in social hierarchy by children, and possibly also by adults. The statistical analysis revealed a significant main effect of body height (OR = 1.9, CI[1.3, 2.7], *p* = 0.001, see Table S1 for full model details; Table 1 for model-predicted results) along with a two-way interaction with age group, suggesting that children and adults weighted the perceptual cue of body height differently (OR = 2.5, CI[1.2, 5.3], *p* = 0.018). Indeed, only children OR = 2.9, CI[1.71,4.99], *p* < 0.001, see Table S7) but not adult participants (OR = 1.2, CI[0.71,2.10), *p* = 0.48, Bayes Factor (BF_01_) = 12.6) based their authority judgements on body height. As shown in Figure 3B, in the children sample, if the index person was the taller person, her probability of being identified as the one in charge increased from 35% to 62% compared to when she was the shorter person. In contrast, the Bayes Factor observed in the adult sample suggests that our findings provide positive evidence in favor of the null hypothesis (i.e., no influence of body height on ascription of authority) as the data are 12.6 times more likely under the null hypothesis than under the alternative hypothesis.

Using the same control models as before, we show that the pattern of results remains qualitatively unchanged when excluding participants with five or more errors in the method check questions, and also across the continuously modeled age range of preschool children (see Tables S2 and S3).

In sum, children judged taller individuals to be higher in authority, while this was not the case for the adult participants.

### The influence of helping behavior on perceived niceness

**Figure 4:**
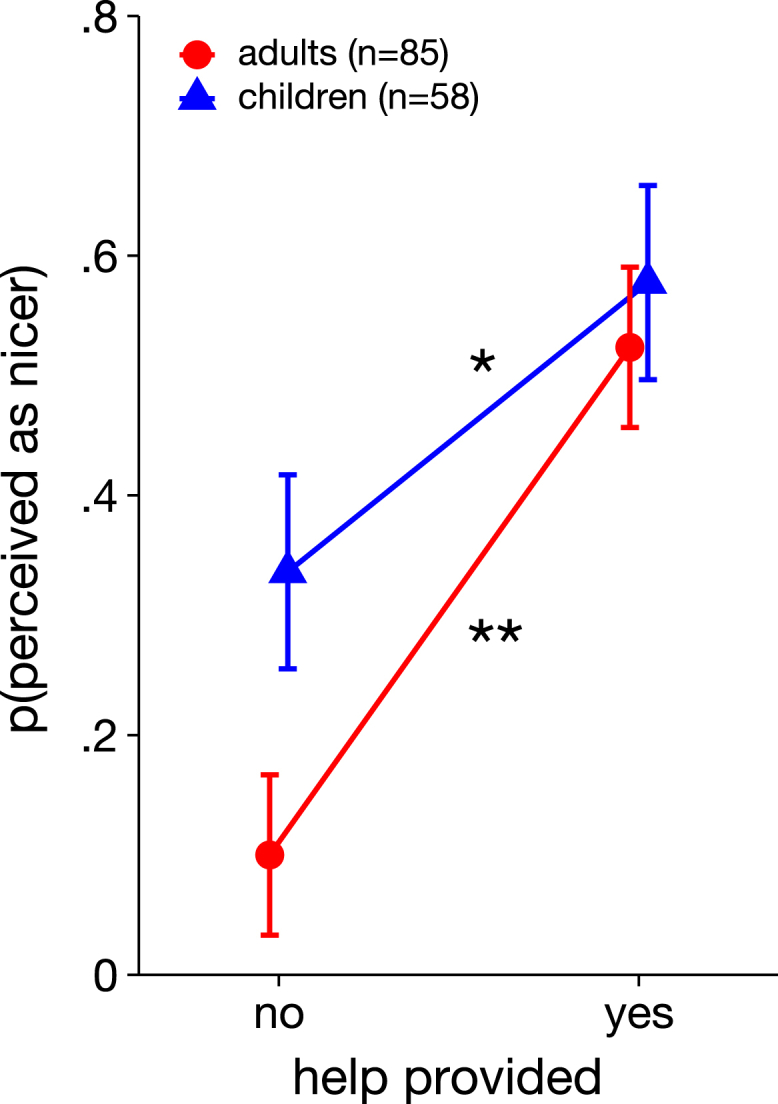
Model-predicted marginal means of perceived niceness as a function of observed behavior and age group As revealed by the significant two-way interaction of helping with age group, children (blue triangle and line) and adults (red circle and line) showed differential weighting of the behavioral cue in their perception of authority: whereas both groups judge helping individuals as being nicer, this effect was more pronounced in the adult sample. Error bars show the 95% confidence interval. ∗ = *p* <0 .01, ∗∗ = *p* <0 .001.

Using a separate mixed-effect model with perceived niceness as dependent variable, we tested our third hypothesis that individuals who provide help are judged to be nicer than individuals who refuse to help, ensuring that participants interpreted the helping scenario as a prosocial act. The model included age group, help, and their interaction as fixed effects. The model explained 16.1% of the total variance. The analysis revealed that the degree to which observed prosocial behavior influenced the perceived niceness differed between the two age groups (OR = 0.83, CI[0.72, 0.97], *p* = 0.016, see Table S4 for full model details; Table 2 for model-predicted results): While both children and adults judged helping individuals as being nicer, this effect was more pronounced in adults (adults: OR = 1.53, CI[1.40, 1.67], *p* <0 .001; children: OR = 1.27, CI[1.12, 1.44], *p* = 0.0015). As shown in Figure 4, the probability of being judged as the nicer one despite not helping was only 10% in the adult sample, and 34% in the children sample. When the index person provided help, the probability increased by 42% for the adults but only by 26% for the children. Children, however, rated the index person more often as the nicer one, irrespective of her helping behavior (OR = 1.16, CI[1.07, 1.25], *p* = 0.001).

**Table 2:** Condition-specific model-predicted probabilities and relative risks for helping behavior and perceived niceness

Predicted probabilities and relative risks (glm)			
*Help*	*Adults p (95% CI)*	*Children p (95% CI)*	*RR (Children/Adults; 95% CI)*
No	0.100 (0.063, 0.155)	0.336 (0.256, 0.427)	3.362 (2.002, 5.646)
Yes	0.524 (0.448, 0.598)	0.578 (0.486, 0.664)	1.103 (0.893, 1.363)

We again, for children only, tested whether the influence of helping behavior on perceived niceness changed across development. Additionally, we also re-ran the main analysis on the reduced sample that excluded participants with five or more errors in the methods check (see Tables S5 and S6). In sum, neither of these additional control variables or their interaction with the main predictor of interest, helping behavior, yielded any significant results, nor did they qualitatively alter the main pattern of results.

In sum, both adults and preschoolers associate prosocial behavior with niceness, which replicates previous findings by Terrizzi et al., 2020.^13^

## Discussion

The present study investigated how body height and (lack of) prosocial behavior influence the perception of authority in adults and preschoolers. While adults attributed more authority to a person who refused to help, we did not find an influence of helping behavior on the ascription of authority in preschoolers. In contrast, preschoolers judged taller individuals as higher in authority, while we did not observe an influence of body height on the authority judgment in adults. As expected, both, children and adults, associated helping behavior with niceness, suggesting that participants understood the content of the video irrespective of age. However, they appear to rely on different cues when attributing social power.

### The link between (refusal of) helping behavior and attribution of authority

In the present study, adults used the absence of helping behavior as a cue for social power, while children did not. Hence, our results confirm prior research suggesting that adults use behavioral cues to attribute authority Terrizzi et al., 2020.^13^ In contrast, preschoolers in the present study did not use refusal to help as an indicator for authority. However, preschoolers did judge a person providing help as nicer, suggesting that they in general understood the scenario, that is, one person helped another and such behavior is generally considered nice.

One explanation for why adults did use refusal to help as indicative of authority while children did not might be the differing everyday experiences of adults and preschoolers. While adults have contact with a large number of diverse authority figures over the course of their lives,^31^ children’s life experience is generally limited to a few already familiar environments.^32^ In their dependence on parents or guardians in the family context, as well as educators in daycare, preschoolers often experience authority figures as those who help in difficult situations, rather than as individuals who refuse to help.

Notably, though, while overall mixed evidence exists as to when prosocial behavior is seen as indicative of authority (see e.g., Hawley, 2002^22^), Terrizzi et al., 2020^13^ did find that preschoolers in a very similar set-up used refusal to help as an indicator for authority. Similar findings were obtained by Thomas et al., 2022,^33^ who could show that in a slightly different set-up, preschoolers expected individuals in power to be less likely to behave prosocially. The difference in results to the present study might arise from a number of factors.

One explanation might be that in addition to helping behavior, we also manipulated body height while Terrizzi et al., 2020^13^ and Thomas et al., 2022^33^ did not. Therefore, when presented with two distinct cues, children might have resorted to the potentially easier to assess cue of body height and neglected difference in helping behavior. Relatedly, variance in body height might have influenced children’s judgment as to who might be capable of helping, as preschoolers have been shown to take into account physical ability when selecting a helper.^26^ If this was the case, the preschoolers in the present study might not have treated helping behavior and body height as independent cues (although we did ensure that, objectively, both shorter and taller actresses would have been capable of providing the necessary help).

Another explanation could be the slightly different wording used in the present study compared to Terrizzi et al., 2020^13^’s study. While Terrizzi et al., 2020^13^ asked their participants “Who is in charge?”, in the present study children were asked “Which of the two is the boss?” (“Wer von den beiden ist die Chefin?”). We chose to use a different wording to facilitate understanding by the preschoolers, since no easy direct translation of “being in charge” exists for German. However, it may have been the case that the resulting focus on the label for an individual (“the boss”) rather than on an attribute or function of that individual (“being in charge”) had an impact on the preschoolers’ responses. However, other studies finding that preschoolers do use behavioral cues to infer authority have also used versions of “Who is the boss?”^24^ making it unlikely that differences in wording explain the present pattern of results.

Finally, differences in location and/or cultural background might have contributed to the difference in results between Terrizzi et al., 2020^13^’s and Thomas et al., 2022^33^’s study and the present findings. While both Terrizzi et al.^13^ and Thomas et al.^33^ conducted their study in a public museum, the children in the present study were visited in their usual daycare centers. Although children were tested in a separate room at the daycare, the overall group context in a daycare setting might have primed a different concept of authority compared to a museum setting, where the child is typically not as part of a group but in most cases with a parent or other adult caretaker. In addition, the daycare system in the United States (where both Terrizzi et al., 2020^13^’s and Thomas et al., 2022^33^’s study was conducted) and Germany (where the present study was conducted) differ sufficiently to be a possible factor.

In sum, the current findings point against the use of refusal to help as a cue for authority in preschoolers and future studies are clearly necessary to address the role of helping behavior or lack thereof with respect to attribution of power across development.

### The link between body height and attribution of authority

The second potential cue to authority investigated in the present study was body height. Here, we observed the reversed pattern compared to helping behavior; while children used body height as indicative of authority, assuming taller people are more likely to be the boss, adults did not rely on body height to attribute authority.

The influence of physical superiority on the perception of social dominance in early childhood^6^^,^^24^ is in line with prior research. In contrast, as Terrizzi et al., 2019^9^ discuss, adults do not exclusively use physical superiority to assess who holds authority (though for instance physical strength does influence attribution of authority also in adults^5^). It is therefore reasonable to assume that adults focus less strongly on external cues in their assessment of social power when other cues such as displayed behavior are available.

An explanation for the finding that children (unlike adults) used body height as a cue might be that for children, body height is a more informative cue for attribution of power in everyday life. In contrast, for adults —at least in modern-day society— this is rarely the case (although some studies suggest a correlation between height and workplace success,^27^ and indeed other studies suggest that also adults associate physical strength with social dominance^34^). First, for children, many authority figures (parents, teachers) are always taller than the child themselves, which is usually not the case in interactions among adults. Second, even among same-aged peers, body height is more predictive of social power compared to an adult setting. Younger children are more likely to solve conflicts via physical confrontation, which a taller child has a larger chance of winning, while the proportion of conflicts solved physically declines with age.^35^

In sum, the results of the present study suggest that adults and children differ in the type of information used to attribute authority. While adults used the (lack of) helping behavior to make statements about a person’s authority, the preschool children were oriented toward the physical size of the actresses.

### Future directions

The present findings suggest a differentiation in cues used to attribute authority by children and adults; while adults relied on display of prosocial behavior, preschoolers used the physical cue body height.

Our findings therefore add to a body of literature painting a complex picture of cue use across development in the determination of social hierarchy. To achieve a clearer understanding, one approach might be to systematically vary combinations of different cues for authority beyond height and helping behavior in different age groups. In particular, this seems essential for reconciling partly conflicting findings, such as between the present results and Terrizzi et al., 2020^13^’s.

Another important future direction to gain a clearer picture might be the use of even more obvious cues. While we used naturalistic stimulus videos (e.g., actresses who naturally differed in body height, and a realistic everyday scenario of a person needing help), exaggerating either factor might facilitate cue interpretation especially for children. This could be achieved by using scenarios in which help is needed more urgently and/or by using computer manipulated video material, in which physical parameters can be better controlled and exaggerated.

Finally, recording not only participants’ judgment but also their looking behavior might yield further insights into how different cues are actually processed. For instance, while children judged a person providing help as nicer, they also perceived the index person overall as nicer, irrespective of her behavior or height. One explanation to be addressed in future studies could be that children paid more attention to the person making decisions (to either help or not help), which could influence their perception of that person. Furthermore, with the present design, we cannot address the question of how an impression of a person evolves over time due to learning effects. With more trials and/or a more nuanced assessment of the participants’ judgment, it would be interesting to investigate to what degree the current judgment of a person is influenced by their prior behavior.

In sum, the present data suggest children and adults ascribe authority based on different indicators; adults relied on displayed helping behavior or refusal thereof, while children relied on body height. Hence, when two conflicting cues are available, adults use the more complex but also more informative one, while preschoolers rely on the more obvious physical cue. Navigating social hierarchies therefore is based on different combinations of cues throughout development.

### Limitations of the study

Like Terrizzi et al., 2020,^13^ we only used female actresses, and cannot rule out the possibility that, for instance, height difference might have a stronger impact when assessing hierarchies among men. Furthermore, we used just two shorter and two taller actresses to record the social interactions. We therefore cannot rule out the possibility that some aspect about their appearance other than height might differentiate taller from shorter actresses. Either a larger number of different videos and actresses or a full combination of actresses across dyads (i.e., each shorter actress paired with each taller actress) or a more nuanced variation via video manipulation as mentioned above could provide further insights here.

Another limitation of the present study is the fact that we provided an explicit definition of *social power* to children but not adults. Hence, we cannot rule out that this definition might have biased the children’s judgment. Note, however that Terrizzi et al., 2020^13^ provided children with a very similar definition and did find an influence of helping behavior on the children’s assessment of social power. Relatedly, we did not assess children’s language skills and hence cannot rule out the possibility that children had a more simplified concept of social power, which might influence their response behavior.

Furthermore, our sample of children included a rather large age range, during which significant development in social skills occurs. Hence, although we did not find an influence of children’s age on their judgment, we cannot exclude the possibility that our sample was too small to capture subtle developmental changes in judgment. Finally, our sample was too small to analyze possible gender-differences in cue use.

## Resource availability

### Lead contact

Requests for further information should be directed to the lead contact Sarah Jessen (sarah.jessen@uni-luebeck.de).

### Materials availability

Two of the stimulus videos can be found on the OSF (https://osf.io/ex7f2/), the other two are available upon request to the lead contact Sarah Jessen.

### Data and code availability

• All data have been deposited at the OSF and are publicly available as of the date of publication. DOIs are listed in the key resources table.
• All original code has been deposited at the OSF and is publicly available as of the date of publication. DOIs are listed in the key resources table.
• No additional information is required to reanalyze the data reported in this paper.

## Acknowledgments

We would like to thank all children for participating and all the preschools for their support, and the DFG for funding to S.J. (JE 781/3-1).

## Author contributions

Conceptualization, S.P., S.W., A.N., and S.J.; investigation, S.P., S.W., and A.N.; formal analysis, S.P., S.W., and S.T.; data curation, S.P., S.W., A.N., and S.T.; writing, S.P., S.W., S.J., and S.T.

## Declaration of interests

The authors declare no competing interests.

## STAR★Methods

### Key resources table

**Table:** 

REAGENT or RESOURCE	SOURCE	IDENTIFIER
**Deposited data**		

Stimulus material	Own	https://osf.io/ex7f2/
Experimental data	Own	https://osf.io/ex7f2/
Analysis code	Own	https://osf.io/ex7f2/

**Software and algorithms**		

R (version 4.2.2)	R Core Team	https://www.r-project.org
TIVIAN	Tivian	https://www.tivian.com/de/

### Experimental model and study participant details

The final sample of children included 58 children between two and six years (*MAge* = 5.45 years [65.4 months], *SDAge* = 1.025 years [12.03 months], min. = 2.5 years [33 months, only one child was younger than 3 years], max. = 6.5 years [78 months], female by parental report = 33). Our sample size was based on the most closely matching prior study,^13^ who included n=64 children in a similar age range and design based on *a priori* power analysis. To guide the reader in their assessment of non-significant effects we additionally report Bayes Factors (see Statistical Analysis).

Children were recruited from seven preschools and daycare centers in Lübeck (Schleswig Holstein - Germany). All legal guardians provided written informed consent for their child’s participation and were informed about the procedure accordingly. In order to participate, children had to have sufficient German language skills and no known neurological disorders, developmental disorders, or non-correctable visual or hearing disorders as reported by their parents. Eight additional children were excluded based on the following reasons: they either did not want to complete the entire experiment (*n* = 2), perceived one of the actresses in the videos as male (*n* = 1), knew one of the actresses (*n* = 1), or failed to pay attention to the videos (*n* = 1). We chose to exclude the child who perceived the actress as male, since we wanted to exclude any potential gender bias on the ascription of authority.^32^ An additional three participants were excluded due to technical problems (*n* = 3). Neither parents nor children received any compensation for their participation.

The adult sample included 85 participants aged between 18 and 57 years who participated in an online version of the experiment (*MAge* = 23.3 years, *SDAge* = 5.62 years, female = 60, male = 23, diverse = 2 [all by self-report]). Recruitment was done via the University of Lübeck email distribution list, social media, and local notices. Most adult participants were university students (*n* = 77). Adults provided written consent by checking the respective online form. Eleven additional subjects had to be excluded for the following reasons: taking an unrealistically short amount of time to complete the online experiment (defined as three standard deviations below the mean; *n* = 2), not watching all videos (*n* = 4), or not passing the method check “Was the tape handed?" (*n* = 5). Students of psychology were offered course credit for their participation.

The study was approved by the ethics committee of the University of Lübeck, Germany (AZ21-507) and conducted according to the declaration of Helsinki. Data collection was carried out between May 12^th^, 2022 and March 1^st^, 2023. Sample sizes were planned based on prior comparable studies, in particular Terrizzi et al. 2020.^13^ We did not perform analyses split up by gender because our sample was not powered for such an analysis.

### Method details

#### Stimulus material

We recorded four videos to be used as stimulus material (Figure 1), each lasting 30 seconds. The videos were closely aligned in content with the videos used by Terrizzi et al., 2020.^13^

Two pairs of actresses were enlisted to record a total of four videos (two per pair). In each pair, one of the actresses was distinctly taller than the other. Each actress wore a t-shirt in a different color to facilitate discrimination. In each of the four videos, the two actresses can be seen standing in a room at approximately the same distance. One of the actresses is trying to mount a poster on a wall. The tape needed for this is lying on a table out of her reach. The actress indicates her inability to access the tape by stretching her arm extensively but without success. There is no verbal request for help in any of the videos. Meanwhile, the second actress (who will be referred to as *index person* in the following) stands a couple of meters away from the actress trying to mount the poster and reads a book. She could easily reach the tape on the table by interrupting her current reading activity. She clearly notices the other person’s problem, which is suggested by a glance to the actress with the poster as well as to the tape. In two of the four videos, the index person decides to put the book down and pass the tape, which enables the other actress to put up the poster. In the other two videos, the index person ends the eye contact and continues reading her book. The poster cannot be mounted successfully.

As mentioned above and in contrast to the videos used by Terrizzi et al., 2020,^13^ we additionally manipulated the factor body height by including a taller and a shorter actress in each pairing. This allowed us to create one video for each of the four experimental conditions as detailed in Figure 2A. The individuals in Figures 1 and 2 have given written informed consent to publish their image.

#### Procedure

The children were tested in their preschool (which is typically attended from about 3 to 6 years in Germany), while adults took part in an online version of the experiment.

For the children, the ten-minute experiment took place in the children’s daycare environment. A quiet room was provided in the respective daycare centers for this purpose. The participation of the child was only possible with the signed consent of the parents, and if the child also agreed to participate. A pair of female experimenters carried out testing for all children.

At the beginning of the experiment, the child was given an age-appropriate definition of a person with social power: *“A person who carries responsibility, makes the rules and tells others what to do.”* After that, the child was shown the four videos in randomized order on a tablet (Samsung Galaxy Tab A8, SM-X205, 10.5” display). After the presentation of each video, the child was shown pictures of the two shown actresses side-by-side on the tablet and asked by the experimenter (1) “*Which of the two is the boss?” (“Wer von den beiden ist die Chefin?”)* and (2) “*Which of the two is nicer?” (“Wer von den beiden ist netter?”)*. The order of the two questions was always the same and the child could respond either verbally or by pointing at one of two actresses shown on the screen. Finally, to ensure that the child had attended to the video and understood the overall scenario, the experimenter asked “*Did one person hand the tape?”* after each video. The child could respond either verbally or by nodding or shaking their head. After these three questions, the next video was presented.

After the experiment, we checked whether the child had been aware of the difference in body height between the actresses. To do so, the child was shown each pair of actresses on the tablet and asked “*Who was taller in the video?”*. Notably, pictures only showed the upper part of the body and were aligned such that the shoulders of both actresses were at the same height. Hence, the child had to remember the height difference from the videos. Finally, the child was again shown all four actresses and asked whether they knew any of them personally.

The adult participants completed the study via the online platform TIVIAN (https://www.tivian.com/de/). Here, instructions were given in written form, and adults were not provided with a definition of social power prior to the experiment. As for the children, adults were shown the four videos in participant-specific random order and asked the same three questions after each video, while seeing a picture of the two actresses (“Which of the two is the boss?”; “Which of the two is nicer?”; “Did one person hand the tape?”). They provided their responses by clicking buttons labeled “the left person”/”the right person” for the first two questions and “yes”/”no” for the third question. In addition, they were asked to judge on an 8-point-Likert scale how certain they were of their judgement regarding authority and niceness. The confidence responses are not analyzed further for the current purpose.

After the experiment, they were asked to indicate who they thought was taller in the videos and whether they knew any of the actresses, comparable to the children.

### Quantification and statistical analysis

For the statistical analysis of responses using logistic regression, we implemented a binary coding scheme: Responses to the questions *Which of the two is the boss?* and *Which of the two is nicer?* were coded as 1 if the index person (i.e., the person who passed the tape or refused to do so) was chosen, and 0 if the other person was chosen. For each of the two questions, a separate generalized (logistic) linear mixed-effect model (GLMM) was computed using the logit link function. Data from children and adults were combined for statistical analysis. Both models included as fixed effects the categorical variables age group (child vs. adult), body height of the index person (shorter, taller [relative to the other person]), and whether help was provided (yes, no). We included participant-specific intercepts as random effects and used simple effect coding for all categorical predictors. The first analysis modeled perceived authority as dependent variable and tested the hypotheses that individuals who (i) refuse to help, and (ii) are taller are judged to be higher in social hierarchy. This model also included the interaction of the two cue manipulations. The second analysis modeled perceived niceness as the dependent variable and tested the hypothesis that individuals who provide help are judged to be nicer. To test whether children and adults weighted the perceptual and behavioral cues (and their potential interaction) differently, we included all possible two-way and the three-way interaction between predictor variables.

For exploratory control analyses, within the sample of children only, we also included the continuously measured age as a regressor. Testing its interaction with the manipulated cue information allowed us to test whether within the included age range there were developmental changes in cue usage. Importantly, we also ran control analyses in which we excluded all participants (N=9) who failed to answer 5 or more method check questions (possible range 0–8). This involved the answers to the questions *Who was taller in the video?,* and *Did one person hand the tape?,* each coded as 0 for correct responses and 1 for incorrect responses.

Note that we used simple (i.e. non-hierarchical) logistic models when mixed-effect model diagnostics suggested singular fits due to insufficient clustering of responses at the level of the individual participants. All models were fit in R (version 4.2.2) using the packages lme4 and sjPlot.

To further guide the interpretability of non-significant results, we calculated the Bayes Factor (BF) based on the Bayes Information Criterion (BIC^36^) extracted from nested models. In detail, the BF_01_ compared the null model (i.e., the model without the effect of interest) to the alternative, more complex model (i.e., including the effect of interest) and indicates how many times more likely the empirical data are under the simpler model. Note that a BF_01_ < 0.1 is interpreted as providing strong evidence against the null hypothesis whereas a BF_01_ > 10 indicates strong evidence for the null hypothesis. The calculation of the Bayes Factor based on the BIC values extracted from the respective estimated models assumes that both the H0 and H1 are equally likely a prior. The null hypothesis (H0) in the present study was that there was no influence of helping behavior/body height on perceived authority, while our research hypothesis (H1) was that helping behavior/body height influenced perceived authority. This approach can be particularly helpful to assess whether a given sample size was simply too small to detect an effect (for a similar approach, see also^37^^,^^38^).
