# Supplementary material for: How do preschoolers and adults ascribe authority?
Source: iScience. 2025 Aug 7;28(9):113279. doi: 10.1016/j.isci.2025.113279 (PMC12396247; doi:10.1016/j.isci.2025.113279)
Supplement: Document S1. Tables S1–S7 [file mmc1.pdf]

iScience, Volume *28*

Supplemental information

How do preschoolers and adults ascribe authority?

Sarah Pieper, Sara Weber, Anna Neuwerk, Sarah Tune, and Sarah Jessen

Supplementary Material

Supplementary Table 1: Predicting perceived authority

	**Perceived Authority [of index person]**			
Predictors	*Odds Ratios*	*CI*	*z*	*p*
(Intercept)	1.689	1.380 – 2.066	5.096	**3.47e-07**
Age group (children)	0.300	0.200 – 0.450	-5.824	**5.73e-09**
Help (yes)	0.475	0.325 – 0.695	-3.830	**1.28e-04**
Body height (taller)	1.894	1.293 – 2.773	3.282	**1.03e-03**
Age group x Help	2.787	1.306 – 5.946	2.650	**8.05e-03**
Age group x Body height	2.502	1.171 – 5.345	2.368	**1.79e-02**
Help x Body height	1.401	0.658 – 2.983	0.875	3.82e-01
Age group x Help x Body height	0.955	0.211 – 4.326	-0.059	9.53e-01
**Random Effects**				
σ^2	3.290			
$\tau_{00\ ID}$	0.123			
ICC	0.036			
N_{ID}	143			
Observations	572			
Marginal R^2 / Conditional R^2	0.175 / 0.205			

Supplementary Table 2: Control analysis of perceived authority (excluding individuals with 5 or more method check errors)

	Perceived Authority [of index person]			
Predictors	*Odds Ratios*	*CI*	*z*	*p*
(Intercept)	1.677	1.358 – 2.070	4.810	**1.51e-06**
Age group (children)	0.296	0.194 – 0.453	-5.617	**1.94e-08**
Help (yes)	0.445	0.299 – 0.664	-3.965	**7.32e-05**
Body height (taller)	1.923	1.290 – 2.869	3.207	**1.34e-03**
Age group x Help	2.442	1.104 – 5.398	2.205	**2.74e-02**
Age group x Body height	2.582	1.165 – 5.721	2.337	**1.94e-02**
Help x Body height	1.207	0.547 – 2.664	0.466	6.41e-01
Age group x Help x Body height	0.710	0.146 – 3.456	-0.425	6.71e-01
**Random Effects**				
σ^2	3.290			
$\tau_{00\ ID}$	0.116			
ICC	0.034			
N_{ID}	134			
Observations	536			
Marginal R^2 / Conditional R^2	0.177 / 0.205			

Supplementary Table 3: Predicted probabilities and relative risks for control analysis model (i.e. excluding participants who made more than 5 errors in the methods check)

Predicted probabilities and relative risks

Help	Height	Adults p (95% CI)	Children p (95% CI)	RR (Children/Adults; 95% CI)
no	shorter	0.852 (0.758, 0.914)	0.385 (0.257, 0.530)	0.451 (0.310, 0.658)
yes	shorter	0.579 (0.469, 0.682)	0.301 (0.187, 0.447)	0.521 (0.323, 0.839)
no	taller	0.852 (0.758, 0.914)	0.657 (0.511, 0.778)	0.771 (0.616, 0.966)
yes	taller	0.663 (0.553, 0.758)	0.573 (0.429, 0.706)	0.865 (0.645, 1.159)

Supplementary Table 4: Predicting perceived niceness

	Perceived niceness [of index person]			
Predictors	*Odds Ratios*	*CI*	*z*	*p*
(Intercept)	0.539	0.441 – 0.655	-6.121	**9.30e-10**
Age group (children)	2.382	1.612 – 3.563	4.300	**1.70e-05**
Help (yes)	5.167	3.505 – 7.743	8.138	**4.02e-16**
Age group x Help	0.273	0.122 – 0.597	-3.217	**1.30e-03**
Observations	572			
R^2 Tjur	0.161			

Supplementary Table 5: Control analysis of perceived niceness (excluding individuals with 5 or more method check errors)

	Perceived niceness [of index person]			
Predictors	*Odds Ratios*	*CI*	*z*	*p*
(Intercept)	0.553	0.449 – 0.678	-5.623	**1.88e-08**
Age group (children)	2.508	1.668 – 3.814	4.369	**1.25e-05**
Help (yes)	5.428	3.620 – 8.274	8.037	**9.21e-16**
Age group x Help	0.301	0.131 – 0.683	-2.850	**4.38e-03**
Observations	536			
R^2 Tjur	0.174			

Supplementary Table 6: Predicted probabilities and relative risks for control analysis model of perceived niceness

Predicted probabilities and relative risks (glm)

**Help**	**Adults p (95% CI)**	**Children p (95% CI)**	**RR (Children/Adults; 95% CI)**
no	0.100 (0.063, 0.155)	0.337 (0.250, 0.436)	3.367 (1.983, 5.719)
yes	0.524 (0.448, 0.598)	0.602 (0.502, 0.694)	1.150 (0.927, 1.427)

Supplementary Table 7: Predicting perceived authority

	Adults sample				Children sample			
Predictors	*Odds Ratios*	*CI*	*z*	*p*	*Odds Ratios*	*CI*	*z*	*p*
(Intercept)	3.299	2.355 – 4.621	6.942	**3.86e-12**	0.926	0.708 – 1.210	-0.561	5.75e-01
Help (yes)	0.267	0.153 – 0.465	-4.651	**3.30e-06**	0.798	0.466 – 1.361	-0.827	4.08e-01
Body height (taller)	1.212	0.707 – 2.077	0.700	4.84e-01	2.902	1.709 – 4.990	3.904	**9.47e-05**
Help x Body height	1.469	0.500 – 4.314	0.700	4.84e-01	1.358	0.466 – 3.976	0.561	5.75e-01
**Random Effects**								
σ^2	3.290							
τ_{00}	0.419 $_{ID}$							
ICC	0.113							
N	85 $_{ID}$							
Observations	340				232			
Marginal R^2 / Conditional R^2	0.110 / 0.210				0.071			
